# Bacterial Vaginosis: Current Diagnostic Avenues and Future Opportunities

**DOI:** 10.3389/fcimb.2020.00354

**Published:** 2020-08-11

**Authors:** Mathys J. Redelinghuys, Janri Geldenhuys, Hyunsul Jung, Marleen M. Kock

**Affiliations:** ^1^School of Clinical Medicine, Wits Reproductive Health and HIV Institute, University of the Witwatersrand, Johannesburg, South Africa; ^2^UP-Ampath Translational Genomics Initiative, Department of Biochemistry, Genetics and Microbiology, Faculty of Health Sciences and Faculty of Natural and Agricultural Sciences, Division of Genetics, University of Pretoria, Pretoria, South Africa; ^3^Department of Medical Microbiology, University of Pretoria, Pretoria, South Africa; ^4^Department of Medical Microbiology, Tshwane Academic Division, National Health Laboratory Service, Pretoria, South Africa

**Keywords:** bacterial vaginosis, female genital tract, diagnostics, vaginal microbiome, vaginal metabolome, vaginal inflammation

## Abstract

A healthy female genital tract harbors a microbiome dominated by lactic acid and hydrogen peroxide producing bacteria, which provide protection against infections by maintaining a low pH. Changes in the bacterial compositions of the vaginal microbiome can lead to bacterial vaginosis (BV), which is often associated with vaginal inflammation. Bacterial vaginosis increases the risk of acquiring sexually transmitted infections (STIs) like human immunodeficiency virus (HIV) and affects women's reproductive health negatively. In pregnant women, BV can lead to chorioamnionitis and adverse pregnancy outcomes, including preterm premature rupture of the membranes and preterm birth. In order to manage BV effectively, good diagnostic procedures are required. Traditionally clinical and microscopic methods have been used to diagnose BV; however, these methods require skilled staff and time and suffer from reduced sensitivity and specificity. New diagnostics, including highly sensitive and specific point-of-care (POC) tests, treatment modalities and vaccines can be developed based on the identification of biomarkers from the growing pool of vaginal microbiome and vaginal metabolome data. In this review the current and future diagnostic avenues will be discussed.

## Introduction

Bacterial vaginosis (BV), or vaginal dysbiosis, is one of the most common vaginal conditions associated with aberrant changes in the vaginal microbiome (VMB) (van de Wijgert et al., 2014). Bacterial vaginosis is characterized by a reduction of the resident lactic-acid producing *Lactobacillus* spp. and an overgrowth of anaerobic bacteria (Hillier et al., 1993; Fredricks et al., 2005; Ravel et al., 2013). This condition poses a major public health concern for women of reproductive age, for their offspring and for their partners as BV is associated with adverse reproductive health outcomes such as pelvic inflammatory disease, miscarriage, preterm birth and may also lead to an increased risk of human immunodeficiency virus (HIV) acquisition and transmission (Hay et al., 1994; Ness et al., 2005; Sha et al., 2005a; Atashili et al., 2008; van de Wijgert et al., 2008).

Bacterial vaginosis can be diagnosed clinically by using the Amsel's criteria (Amsel et al., 1983) and in the laboratory using the Nugent scoring system (Nugent et al., 1991). Amsel's criteria were published in 1983 for use in clinical settings and at least three of four criteria need to be met before being classified as BV positive (Amsel et al., 1983). The Nugent scoring system is a refined version of the grading criteria introduced by Spiegel et al. (1983), where Gram-stained vaginal smears are evaluated for the presence/absence and quantity of specific bacterial morphotypes with a scoring system ranging from 0 to 10 (Nugent et al., 1991).

Although both methods have been widely used worldwide for almost three decades and are considered the “gold standard” of BV diagnosis, these methods are not free of limitations. For example, both methods are often subject to interobserver variability as the assessment of the diagnostic criteria depends on the observer's skill and experience (Klebanoff et al., 2004; Modak et al., 2011). Inaccurate BV results may lead to misdiagnosis and delays in treatment, which eventually puts women at risk for adverse reproductive health sequelae (Allsworth and Peipert, 2007; Modak et al., 2011). Misdiagnosis may also occur because of the unavailability of specific diagnostic tools in e.g., resource-limited settings and the deviation from strict diagnostic criteria by clinicians (Chavoustie et al., 2017). The complex etiology of BV contributes to inaccurate diagnosis, especially in asymptomatic carriers, and subsequently to poor treatment and clinical outcomes. The continuous development of accurate, easy-to-use point-of-care (POC) tests for BV is crucial, particularly in resource-limited settings.

The major drawbacks for the development of new diagnostic assays for BV are the lack of a uniform case definition for BV and that the etiology of BV remains poorly understood (Forsum et al., 2005; Muzny and Schwebke, 2016). In recent years, the advancement of molecular and high-throughput sequencing technologies [e.g., next-generation sequencing (NGS)] has revealed that BV is a multifactorial condition influenced by social, epidemiological, microbiological and host factors (Muzny and Schwebke, 2016; Muzny et al., 2020). Also, evidence suggests that the presence of a polymicrobial biofilm offers protection for many BV-associated bacteria against hydrogen peroxide (H_2_O_2_), antibiotics and host immunity (Swidsinski et al., 2005, 2008, 2015; Patterson et al., 2007) and these factors should be considered when designing a POC test. The focus of this review includes factors that complicate the diagnosis of BV, currently available diagnostic avenues and alternative approaches as potential diagnostic avenues for the diagnosis of BV.

## Factors Complicating the Diagnosis of BV

The diagnosis of BV is complicated by the lack of consensus over what is defined as BV, the natural variation of the VMB in women of different racial backgrounds and the elusive polymicrobial etiology of BV. Several risk factors have been identified in the pathogenesis of BV, such as age, socioeconomical status, antibiotic usage, sexual behavior and ethnicity (Brumley, 2012; Singh et al., 2015; Ranjit et al., 2018). *Lactobacillus* bacteria are traditionally linked with a healthy VMB, but *L. iners* may be more pathogen than friend. These factors remain the reason that still no one method can accurately diagnose BV.

### The Definition and Pathogenesis of BV

Historically, “non-specific bacterial vaginitis” was defined by Gardner and Dukes (1954) as an “infection” caused by a single etiological agent, *Haemophilus vaginalis* (now renamed as *Gardnerella vaginalis*), based on the observation that this bacterium was isolated from 92% (127/138) of women with “non-specific bacterial vaginitis.” In the following year, the authors argued that the direct inoculation of *G. vaginalis* in women “free of the disease” resulted in clinical manifestations of BV and pure cultures of *G. vaginalis* could be recovered from these women (Gardner and Dukes, 1955). However, in the very same study, this argument was not true in the majority of the tested “disease-free” women (77%; 10/13), and positive cultures were not obtained from these women (Gardner and Dukes, 1955). In confirmation of these findings, studies based on molecular and culture techniques have shown that *G. vaginalis* can be found in the vaginal tract of sexually inexperienced women and of women without BV (Nugent score 0–3) (Aroutcheva et al., 2001; Fethers et al., 2012; Balashov et al., 2014; Schwebke et al., 2014a; Janulaitiene et al., 2017). Consequently, the “single etiological agent” theory was not widely accepted (Hickey and Forney, 2014).

To address the controversy of the “single etiological agent” concept, alternative definitions of BV suggested that this condition is of polymicrobial nature. There are several proposed polymicrobial hypotheses under debate, mainly to answer three questions: (i) whether BV is initiated by the establishment of an “early colonizer species” like virulent strains of *Gardnerella* spp. and *Prevotella bivia*, which create a favorable vaginal environment to facilitate the adherence and the growth of the “secondary colonizer” species like *Atopobium vaginae* or *Megasphaera* type I; (ii) whether BV is initiated by the introduction of polymicrobial communities of BV-associated bacteria; or (iii) whether the displacement of *Lactobacillus* spp. happens before the establishment of “early colonizer species” or polymicrobial BV communities (Srinivasan and Fredricks, 2008; Schwebke et al., 2014b; Muzny et al., 2018, 2019). In addition, it is also unclear whether BV is caused by *de novo* polymicrobial biofilm formation or by the transmission of polymicrobial biofilms through sexual activity (Verstraelen and Swidsinksi, 2019). While there is some evidence supporting each hypothesis, these hypotheses explaining the polymicrobial BV theory have not come to a consensus yet. In support of the first question above, a study by Pybus and Onderdonk (1997) suggested that during the onset of BV, *Gardnerella* spp. may provide a favorable environment by producing amino acids to promote the growth of other BV-associated bacteria (e.g., *P. bivia*). Muzny et al. (2018) showed that the mean relative abundance of four “key bacteria” (i.e., *P. bivia, Gardnerella* spp., *A. vaginae* and *Megasphaera* type I) is significantly increased zero to four days before the onset of BV, indicating their role during the initiation of BV. However, relative abundances of certain *Gardnerella* spp. are not always associated with BV as no significant association was found between relative abundances of *G. leopoldii* and Nugent scoring category by Hill et al. (2019). *Gardnerella leopoldii* is a recently-identified species within the genus *Gardnerella* and is part of 13 genomic species found within the genus through whole genome sequence analysis and matrix-assisted laser desorption ionization time-of-flight mass spectrometry (MALDI-TOF MS) analysis (Vaneechoutte et al., 2019). With regards to the second question above, Srinivasan and Fredricks (2008) have hypothesized that BV is triggered when the polymicrobial communities of BV-associated bacteria are introduced into the vaginal environment, which may lead to simultaneous displacement of lactobacilli. In support of this hypothesis, there is evidence that some strains of BV-associated bacteria (*Bacteroides* spp., *Enterococcus faecium, G. vaginalis, Mobiluncus* spp., and *Peptostreptococcus* spp.) are capable of inhibiting the growth of lactobacilli (Nagy et al., 1991; Kelly et al., 2003). Furthermore, according to a study by Swidsinski et al. (2014), the polymicrobial community of BV-associated bacteria could be transmitted by means of a polymicrobial biofilm (“clue cells,” characteristic vaginal epithelial cells coated with coccobacillary bacteria). This hypothesis, however, is contradicted by the studies that suggest other BV-associated bacteria are not as virulent as *Gardnerella* spp. in terms of cytotoxicity, adherence and biofilm formation (Patterson et al., 2010; Machado et al., 2013a; Alves et al., 2014).

Despite multiple molecular and genomic studies being conducted at species or microbiome level, there is no consensus on the group of bacterial species that may directly cause BV (Fethers et al., 2012; Srinivasan et al., 2012; Chen et al., 2018; Muzny et al., 2018). Srinivasan et al. (2012) performed broad-range 16S ribosomal RNA (rRNA) gene PCR and pyrosequencing to describe the composition and diversity of the VMB in women with BV, diagnosed either by Amsel's criteria or Nugent scoring. The results revealed that women with BV have a heterogeneous VMB that is not dominated by a single taxon and no species is universally present in all of BV-positive women (Srinivasan et al., 2012). It is supported by the findings of Chen et al. (2018) that significant differences in the bacterial composition of the BV microbiome were observed among BV groups defined by Amsel's criteria or Nugent scoring.

Vaginal inflammation is another controversial feature in BV. Some studies have reported that there was no statistically significant difference between any of the median concentrations of the pro-inflammatory cytokines [interleukin 6 (IL-6), IL-10, IL-12] tested for in BV-positive women vs. controls, and that high levels of prolidase and sialidase have led to the cleavage of the vaginal immunoglobulin A (IgA) and IgM (Cauci et al., 1998; Weissenbacher et al., 2010). A recent study using a mouse coinfection model also found that neither *Gardnerella* spp. or *Prevotella bivia* induced vaginal inflammation (Gilbert et al., 2019). In contrast, other studies have provided evidence that vaginal inflammation might well be present in BV and that specific BV-associated bacteria other than *Gardnerella* spp. and *Prevotella* spp. may induce proinflammatory responses (Sturm-Ramirez et al., 2000; Hedges et al., 2006; Nikolaitchouk et al., 2008; Anahtar et al., 2015; Jespers et al., 2017; Gardner et al., 2019; Gilbert et al., 2019). A few examples include a study by Sturm-Ramirez et al. (2000), in which women with BV had higher odds of having high levels of IL-1β or a tumor necrosis factor alpha (TNF-α) (adjusted odds ratio: 4.17; 95% confidence interval: 1.69–10.48; *p* = 0.002) and a study by Jespers et al. (2017), in which statistically significant increased concentrations of IL-12 were observed in women with incident BV. Similar observations were reported regarding other types of proinflammatory cytokines (IL-1α, IL-8, IL-36γ) (Pivarcsi et al., 2005; Nikolaitchouk et al., 2008; Gardner et al., 2019). Accordingly, a conceptual model was recently proposed by Muzny et al. (2020) in order to explain these observations. According to this model, epithelial cells and immune cells during a “healthy state” contribute to homeostasis by producing anti-inflammatory cytokines in response to low levels of cytokines produced by epithelial cells, whereas during early colonization by *Gardnerella* spp. and *Prevotella bivia*, sialidase is produced to induce a mucosal barrier disruption and allow the evasion of immune responses (Lewis et al., 2012; Gilbert et al., 2019; Muzny et al., 2020). Ultimately, secondary colonizers like *A. vaginae* and *Sneathia* spp. may join the polymicrobial biofilm and elicit the production of proinflammatory cytokines (Libby et al., 2008; Anahtar et al., 2015; Muzny et al., 2020). However, more research needs to be done to elucidate exact mechanisms of how the host-microbiota interactions contribute toward BV etiology.

### Variation in the Vaginal Microbiome

Bacterial vaginosis has a complex pathogenesis and etiology and a disruption in the vaginal microflora is thought to be the main contributor to the altered vaginal environment and the associated clinical symptoms (Muzny and Schwebke, 2016). However, up to 50% to 75% of BV cases could be asymptomatic, complicating the diagnosis of BV and raising more questions on the etiology of this condition (Klebanoff et al., 2004; Coleman and Gaydos, 2018).

Traditionally, the vaginal environment of “healthy” women (referring to women who do not have adverse reproductive health outcomes and do not show clinical symptoms) were thought to be protected and maintained by hydrogen peroxide (H_2_O_2_)-producing *Lactobacillus* spp. *via* the production of H_2_O_2_, lactic acid and bacteriocins (Klebanoff et al., 1991; Zhou et al., 2004; O'Hanlon et al., 2011; Ravel et al., 2011). A lack or a decrease in the number of *Lactobacillus* spp. was therefore considered to indicate vaginal dysbiosis and this assertion formed the basis for the Nugent scoring system, which regards the lack of the *Lactobacillus* morphotype and the abundance of Gram-variable rods and cocci (*G. vaginalis* and *Bacteroides* spp. morphotypes) as BV (Nugent score of 7–10; Klebanoff et al., 1991; Nugent et al., 1991; Hillier et al., 1993). Indeed, a large cross-sectional study in the US by Ravel et al. (2011) showed that the VMB of 396 “healthy” women [representing four different ethnic groups (white, black, Hispanic, and Asian)] consisted of five “community state types” (CSTs), in which four CSTs were dominated by four *Lactobacillus* species each (*L. crispatus, L. gasseri, L. iners* and *L. jensenii*) (72.2%; 286/396). However, women with more heterogeneous CST (dominated by strict BV-associated anaerobic bacteria such as *Prevotella* spp., *Dialister* spp., *Atopobium* spp., *Gardnerella* spp., *Megasphaera* spp., *Peptoniphilus* spp., *Sneathia* spp., *Eggerthella* spp., *Aerococcus* spp., *Finegoldia* spp., and *Mobiluncus* spp.) were asymptomatic and therefore thought to still maintain a “healthy” vaginal environment (Ravel et al., 2011). This type of VMB was also observed in several other studies (Zhou et al., 2004; Hyman et al., 2005; Fettweis et al., 2014; Gautam et al., 2015). The reason why women with this CST can still maintain “healthy” vaginal environments is yet unknown, but it is possible that anaerobic bacteria like *Atopobium* spp., *Leptotrichia* spp. and *Megasphaera* spp. may substitute an ecological role in the absence of *Lactobacillus* spp. by e.g., producing lactic acid (Zhou et al., 2004). This substitution of an ecological role by anaerobic bacteria may partially explain why there is a high percentage of asymptomatic BV and may also contribute to the misdiagnosis of BV (Hickey et al., 2012). Therefore, with more information coming to light, the diagnosis of BV should not be focused on only the abundance of lactobacilli and other anaerobic bacteria.

The diagnosis of BV could be complicated by the fact that the vagina is a dynamic ecosystem that undergoes a natural fluctuation in the composition of the VMB throughout a woman's life, which is influenced by menstrual cycle, progesterone and estradiol levels, glycogen content in the vaginal epithelium, vaginal pH and immune responses (Brotman et al., 2010; Thoma et al., 2011; Gajer et al., 2012; Jespers et al., 2017; De Seta et al., 2019; Gliniewicz et al., 2019). At birth, it is believed that the VMB is established when the neonate is exposed to the vaginal tract of her mother during vaginal delivery or to the skin bacteria during Caesarian section (Dominguez-Bello et al., 2010). During this time, the VMB of the neonate delivered through the vaginal tract resembles that of her mother, dominated by either *Lactobacillus* spp., *Prevotella* spp. or *Sneathia* spp. (Dominguez-Bello et al., 2010). When the neonate gets older, the vaginal epithelium becomes thinner (i.e., contains a lower glycogen content) and the vaginal pH becomes neutral due to the decreased number of *Lactobacillus* spp. producing lactic acid (Farage and Maibach, 2006a). These physiological changes trigger the established VMB to undergo a compositional change from a *Lactobacillus* spp.-dominated VMB to a VMB dominated by strict anaerobes (e.g., *Bacteroides fragilis*) or enteric bacteria (e.g., *Escherichia coli*; Hammerschlag et al., 1978a,b). The vaginal pH and the number of *Lactobacillus* spp. seem to recover in adolescents and in women of reproductive age as the vaginal epithelium thickens (i.e., the glycogen level rises) under the estrogen influences (Eschenbach et al., 2000; Alvarez-Olmos et al., 2004; Farage and Maibach, 2006b). At this time (reproductive age), the VMB can undergo a natural fluctuation in composition during the menstrual cycle—some women may have a more resilient VMB while others have more extensive fluctuation over short periods of time (Gajer et al., 2012).

The role of ethnicity and race in affecting patterns of the vaginal microbiome have been considered because of the higher prevalence of BV in African-American and Mexican-American women (51.5 and 32.1%, respectively) in comparison to white, non-Hispanic women (23.2%; Alcendor, 2016). Some women (especially of African ethnicity) may also harbor a more heterogeneous VMB consisting of strict anaerobes like *Anaerococcus* spp., *Atopobium* spp., *Corynebacterium* spp., *Finegoldia* spp., *Gardnerella* spp., *Megasphaera* spp., *Prevotella* spp. and *Streptococcus* spp., along with *Lactobacillus* spp. (Zhou et al., 2007; Gajer et al., 2012; Fettweis et al., 2014). This type of VMB is similar to the VMB observed in postmenopausal women, in which the numbers of *Lactobacillus* spp. are reduced but those of strict anaerobes like *Anaerococcus* spp., *Atopobium* spp., *Finegoldia* spp., *Gardnerella* spp., *Prevotella* spp. and *Streptococcus* spp. are increased (Brotman et al., 2018; Gliniewicz et al., 2019).

### The Role of *Lactobacillus* Iners in the Diagnosis of BV

The VMB is typically characterized by the dominance of a single or a few *Lactobacillus* species such as *L. crispatus, L. gasseri, L. jensenii, L. iners* and to a lesser extent species such as *L. acidophilus, L. brevis, L. delbrueckii, L. fermentum, L. mucosae, L. paracasei, L. plantarum, L. reuteri, L. rhamnosus* and *L. vaginalis* are also VMB colonizers (Antonio et al., 1999; Pavlova et al., 2002; Tärnberg et al., 2002; Ravel et al., 2011). If characterization of the VMB is considered as part of the diagnostic criteria for BV, understanding the relevance and functional role of different *Lactobacillus* species is needed. The identification of different types of lactobacilli in the VMB may be relevant to understand the stability of the VMB and the host's susceptibility to pathogens (Spear et al., 2011). *Lactobacillus iners* has been detected in the VMB of both BV positive and BV negative women, raising questions about the role of *L. iners* in the etiology of BV (Macklaim et al., 2011; Petrova et al., 2017). While the detection of *G. vaginalis* and *A. vaginae* have received the most attention in the etiology of BV, *L. iners* has been suggested as an unusual suspect in the etiology of BV and might play a critical role in the diagnosis of BV (Vaneechoutte, 2017).

The predominance of *L. iners* in an intermediate VMB or in symptomatic and asymptomatic BV might indicate its role in homeostasis and in promoting a lactobacilli-dominated microbial community in an altered vaginal environment (Ferris et al., 2004; Jakobsson and Forsum, 2007; Lambert et al., 2013). High levels of *L. iners* in BV may also refer to its genetic composition enabling optimal adaptation and survival in altered vaginal environments (Petrova et al., 2017). For instance, functional analysis in cases of BV revealed the expression of genes for the breakdown of glycogen, mannose and maltose (Macklaim et al., 2013). Shipitsyna et al. (2013) highlighted the importance of *L. iners* after finding *L. iners* and *G. vaginalis* as the predominant species in intermediate cases of BV. Another finding in the same study was the decline in the abundance of *L. iners* in cases of BV as opposed to levels in healthy women. The depletion of the abundance of *L. iners* together with the possibility that the predominance of *L. iners* may shift an intermediate VMB further toward dysbiosis contribute to the value of *L. iners* in the diagnosis of BV (Verstraelen et al., 2009; Petrova et al., 2017).

In the study by Shipitsyna et al. (2013), the qualitative detection of *L. iners* (negative result) was determined to have a low sensitivity in the prediction of BV (7% sensitivity and 86% specificity) with also little discriminatory power between positive and negative BV cases. Microscopy techniques such as Gram-staining may also lead to false negative results due to different isolates of *L. iners* presenting with different bacterial morphologies than that previously described (De Backer et al., 2007; Petrova et al., 2017). Thus, far, the most reliable method to include *L. iners* in the diagnosis of BV would be a quantitative method, including sequencing of the 16S rRNA gene that has previously enabled a comparison of prevalence, bacterial load and relative abundance of BV associated bacteria (Shipitsyna et al., 2013). Together with the relative abundance of *L. iners*, different ethnic groups with known differences in the abundance of *L. iners* in the VMB should also be considered when BV is suspected. Especially in cases involving Black African and African-American women where *L. iners* has been strongly associated with symptomatic and asymptomatic cases of BV (Ravel et al., 2011; Jespers et al., 2012; Srinivasan et al., 2012; Mitchell et al., 2013; Vaneechoutte, 2017).

## Current Diagnostic Avenues

Bacterial vaginosis is clinically characterized by Amsel's criteria or laboratory diagnosed by the Nugent score (Bautista et al., 2017). Clinical laboratories identify changes in the vaginal environment through microscopic examination and vaginal swab culture (Hong et al., 2016). Nugent scoring involves Gram-staining of vaginal smears and has been suggested as the gold standard in the diagnosis of BV in comparison to Amsel's criteria, which is based on non-quantifiable, non-reproducible clinical symptoms only (Chawla et al., 2013; Amegashie et al., 2017; Antonucci et al., 2017; Coleman and Gaydos, 2018). The diagnosis of BV is made on quantification of the Gram-stained microorganisms, classifying these organisms based on different vaginal morphotypes as well as the identification of clue cells (part of the Amsel's criteria) which could be laborious and requiresskilled personnel (Nugent et al., 1991; Chawla et al., 2013; Antonucci et al., 2017).

Amsel's criteria involves saline microscopy and has been improved over time to include the presence of a thin watery homogenous discharge, elevated vaginal pH (>4.5), the presence of more than 20% of clue cells (vaginal epithelial cells) and a fishy odor after the addition of 10% potassium hydroxide to vaginal secretions (“whiff test”) for a positive BV diagnosis (Amsel et al., 1983; Eschenbach et al., 1988; Mohammadzadeh et al., 2014). In comparison to Nugent scoring, the sensitivity and specificity for Amsel's criteria ranges from 37% to 70% and 94% to 99%, respectively and with moderate reproducibility (Schwebke et al., 1996; Sha et al., 2005b). Previous studies determined that 37% to 54% of women with an intermediate Nugent scoring had BV according to the Amsel's criteria (Taylor-Robinson et al., 2003; Bradshaw et al., 2005). A combination of Amsel's criteria and Nugent scoring may be beneficial for an accurate diagnosis of BV due to an assessment on both clinical symptoms and microbial morphology.

Another diagnostic method based on Gram-stained vaginal smears, the Ison-Hay classification criteria was described in 2002, which allows simplified grading and characterization of the vaginal microflora based on the amount of lactobacilli morphotypes compared to *Gardnerella* spp. morphotypes (Ison and Hay, 2002; Chawla et al., 2013). A vaginal microflora with a Nugent score of 4 to 6, known as an intermediate score or intermediate microflora is classified in grade II by the Ison-Hay criteria (Amegashie et al., 2017; Antonucci et al., 2017). An intermediate microflora was initially thought as a transitional step between a normal vaginal microflora and BV, or *vice versa*, but remains an uncharacterized category and is a challenge in the diagnosis of BV due to unknown clinical implications (Verhelst et al., 2005; Menard et al., 2008). Microscopy may be desired by some clinicians above other laboratory tests such as molecular assays due to a shorter turn-around time; however, the identification of different morphotypes is subjective and diagnosis may be influenced by individual skills and experience (Chawla et al., 2013; Antonucci et al., 2017). The involvement of species such as *A. vaginae, Ureaplasma* spp. and *Mycoplasma* spp., that cannot be detected by using Gram staining techniques or Nugent score, subsequently decreases the sensitivity of the Nugent score and warrants the need of a confirmatory or an additional molecular test to measure other etiological agents in the diagnosis of BV (Menard et al., 2008; Haggerty et al., 2009).

Several POC diagnostic assays exist to diagnose BV, such as saline microscopy, wet mount microscopy, chromogenic tests such as the OSOM® BVBlue® and the VGTest™ ion motility spectrometry (IMS). The VGTest™ IMS determines the levels of the malodorous biogenic amines associated with BV, whereas the OSOM® BVBlue® test detects elevated levels of the sialidase enzyme to diagnose BV (Madhivanan et al., 2014; Blankenstein et al., 2015). Rapid assays detecting the presence of proline amino peptidase in BV have also demonstrated high levels of specificity and sensitivity (Madhivanan et al., 2014). The combination of testing for vaginal pH and the whiff test is also suggested as a simple and inexpensive POC test for especially resource-limited settings (Madhivanan et al., 2009). The development of nucleic acid amplification tests (NAATs) such as the BD Affirm™ VPIII test can identify and differentiate between organisms associated with vaginitis and should be investigated to be used as a POC test (Cartwright et al., 2013).

### Problem of Syndromic Management in Resource-Limited Settings

Syndromic management is based on the identification of a combination of symptoms presented during a clinical examination (Shrivastava et al., 2014). These symptoms may be easily recognized signs associated with infection and known bacterial pathogens (Shrivastava et al., 2014). A diagnosis is made by a healthcare provider within a short time, without sophisticated skills and laboratory tests (Altini, 2006). Syndromic management of sexually transmitted infections (STIs) and BV has its benefits, especially in resource-limited settings where invasive procedures and laboratory tests are not available. These benefits include the amendment of immediate treatment on the patient's first visit to a clinic, (reducing further transmission of the disorder), it's widely accessible, education is provided, and no laboratory tests are needed (World Health Organization, 2007; Shrivastava et al., 2014). The success of syndromic management relies on accurate information including the sexual history of the patient and a thorough clinical examination (National Department of Health (South Africa), 2015). Bacterial vaginosis is diagnosed based on vaginal discharge syndrome (VDS) and is treated with a stat dose of metronidazole (National Department of Health (South Africa), 2015).

Despite the advantages that have been reported with syndromic management of STIs and BV, the shortfall of this approach includes not detecting asymptomatic STIs (that may be under treatment) and a poor positive predictive value where antibiotic susceptibility testing is not available, possibly resulting in the overuse of antibiotics (Garrett et al., 2018). In resource-limited settings, there are limited opportunities for routine surveillance, complicating the treatment and resolution of infections. Deviation from standard management guidelines by healthcare workers and the habit of relying on their own clinical judgement also contributes to insufficient treatment of vaginal infections (Leitich et al., 2003; Tann et al., 2006).

Syndromic management may lead to the misdiagnosis of BV and treatment failure and high rates of recurrencemay contribute to antibiotic resistance (Bostwick et al., 2016). A study conducted in South Africa investigating the prevalence of asymptomatic BV amongst a group of HIV unaffected pregnant women observed that 43% of BV positive (severe BV as indicated by a Nugent score of 9–10) women were asymptomatic (Joyisa et al., 2019). The high number of asymptomatic BV cases complicates syndromic management. Diagnosis and effective treatment of BV in pregnant women are warranted to avoid the development of pregnancy complications as well as the acquisition of HIV and other STIs. To diagnose and treat asymptomatic BV, low-cost but sensitive and specific diagnostic models for resource-limited countries are needed in addition to a proposed shift from syndromic to diagnostic management (Shrivastava et al., 2014; Garrett et al., 2018).

## Alternative Approaches as Potential Diagnostic Avenues for the Diagnosis of BV

Rapid and accurate molecular methods targeting the nucleic acid have changed the diagnosis of BV and also provided new insight into our understanding of BV. The report that *Gardnerella vaginalis* is not just one species and does not only comprise one strain type but that several genomic species can be distinguished in the *Gardnerella* genus (Vaneechoutte et al., 2019) has emphasized how limited our understanding of bacterial vaginosis is and how the use of current and advanced technologies will shape our understanding of this condition in the future. Next generation technologies, including genomics, metabolomics, proteomics and immunomics shed light on the functional and immune processes during healthy, intermediate and BV states, that can ultimately contribute to the development of diagnostic tests, treatment and prevention strategies. The fourth industrial revolution brings about artificial intelligence and the possibility of diagnosing complex syndromes, like BV, using machine learning algorithms.

### Molecular Diagnostic Methods

**Figure 1:**
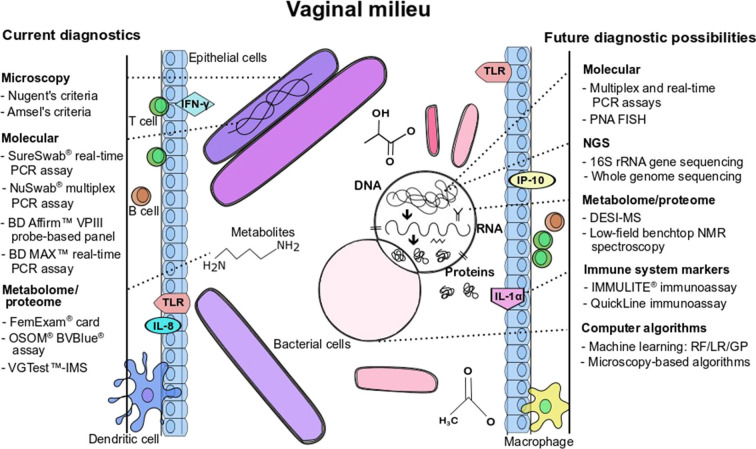
Current diagnostics and future possibilities for BV detection and/or BV pathogenesis characterization. Figure annotations in acronym or abbreviated form are: DESI-MS, desorption electrospray ionization-mass spectrometry; GP, genetic programming; IFN, interferon; IL, interleukin; LR, logistic regression; NMR, nuclear magnetic resonance; PCR, polymerase chain reaction; PNA-FISH, peptide nucleic acid fluorescence *in situ* hybridization; RF, random forest; TLR, toll-like receptor; VGTest-IMS, VGTest-ion mobility spectrometry.

Given the limitations of Amsel's clinical criteria and microscopy for the diagnosis of BV, the development of molecular assays is an attractive approach for the diagnosis of BV because of the ability to identify and quantify multiple fastidious microorganisms (Adzitey et al., 2013). As described by Coleman and Gaydos (2018), an example of a molecular technique used in the diagnosis of BV is the direct probe assay (BD Affirm™ VPIII probe assay) that is commercially available (Becton Dickinson, Sparks, MD) and the Bacterial Vaginosis/Vaginitis panel developed by Quest diagnostics. Direct probe assays are rapid and can detect multiple indicator organisms in a single sample but may require high concentrations of the targeted bacteria for a reliable result. The detection of only *G. vaginalis* in direct probe assays is not a specific marker for BV (thus cannot be used to diagnose BV), as this species may be present in healthy women. The specific target used in these probe assays should be verified as 13 genomic species have been identified in the genus *Gardnerella* (Vaneechoutte et al., 2019). Other clinical indicators such as Amsel's criteria could be used in conjunction with probe assays to improve diagnostic accuracy and ensure reliable diagnosis (Spiegel, 1991; Fredricks et al., 2005; Zozaya-Hinchliffe et al., 2010; Janulaitiene et al., 2017). Direct probe assays designed for the detection of *G. vaginalis* might be more specific for the diagnosis of BV in symptomatic patients than in asymptomatic patients. A sensitivity and a specificity of 90% and 97%, respectively have been reported when compared to microscopy and Nugent scoring (94% and 81%, respectively; Coleman and Gaydos, 2018). Nucleic acid amplification tests are another molecular approach that is proven to have a higher diagnostic accuracy (20% to 25%) over direct probe assays, especially in populations with varying prevalence of infective vaginitis (Cartwright et al., 2013). Commercially available NAATs as indicated in Table 1 include the NuSwab® quantitative multiplex PCR assay, SureSwab BV DNA quantitative real-time PCR assay, BD Max vaginal panel and the BV multiplex assay with sensitivity ranging from 90.5% to 96.7% (the latter for symptomatic women) and specificity from 85.8% to 95% compared to Amsel's criteria and Nugent score as previously reported in detail by Coleman and Gaydos (2018). Figure 1 summarizes the main diagnostic tests and/or approaches that are currently available in the diagnostic arena for BV detection and approaches that could be developed for wider use in BV diagnostics.

**Table 1:** Commercially-available tests for BV, with diagnostic capability and markers of detection.

**Test and manufacturer**	**Quantitative or qualitative**	**BV markers**
NuSwab® (LabCorp, Burlington, NC)	Semi-quantitative; Qualitative	*Atopobium vaginae*, BVAB2, *Megasphaera-1, L. crispatus* (Cartwright et al., 2012)
SureSwab® real-time PCR assay (Quest diagnostics, Secaucus, NJ)	Quantitative; Qualitative	*Lactobacillus* spp., *Atopobium vaginae, Megasphaera* spp., *Gardnerella vaginalis*
BD MAX™ real-time PCR assay (Becton, Dickinson and company, Franklin Lakes, NJ)	Quantitative; Qualitative	BV Candida group (*C. albicans, C. tropicalis, C. parapsilosis*, and C. dubliniensis), *C. glabrata* or *C. krusei*, and *Trichomonas vaginalis, Lactobacillus* spp. (*L. crispatus, L. jensenii, Atopobium vaginae, Megasphaera* spp., *Gardnerella vaginalis*, BVAB2 (Gaydos et al., 2017)
BV multiplex assay (Medical Diagnostic Laboratories, Hamilton Township, NJ)	Quantitative; Qualitative	*Lactobacillus* profiling *Atopobium vaginae, Megasphaera* phylotypes 1 and 2, *Gardnerella vaginalis*, BVAB2 (Hilbert et al., 2016)
OSOM® BVBlue® assay (Gryphus Diagnostics, LLC, Knoxville, TN)	Qualitative	Sialidase enzyme activity (Myziuk et al., 2003)
FemExam® card (Litmus Concepts, Santa Clara, CA)	Qualitative	Vaginal pH and amine activity (West et al., 2003)
IMMULITE® immunoassay (Siemens Healthcare, Erlangen, Germany)	Quantitative; Qualitative	Levels of cytokines: TNF-α, IL-6, IP-10 (Zirath et al., 2017)
QuickLine immunoassay (Milenia Biotec, Gießen, Germany)	Quantitative; Qualitative	Levels of cytokines: IL-6, TNF-α (Kunze et al., 2016)

Nucleic acid amplification tests are dependent on the selection of specific indicator organisms; however, multiplexed NAATs allow the detection of multiple indicator organisms in a single NAAT reaction (Adzitey et al., 2013). Not only is the selection of indicator organisms, but also the inclusion of the quantitative value that would indicate an accurate BV diagnosis, a challenge. Indicator species such as *G. vaginalis* and *A. vaginae* can also be commensals of a healthy VMB (Zozaya-Hinchliffe et al., 2010; Redelinghuys et al., 2017). The value of molecular quantification in the diagnosis of BV was investigated by Menard et al. (2008) who, similar to other studies, investigated the bacterial load to determine cut-off values for both *G. vaginalis* and *A. vaginae* (Wang and Su, 2014; Redelinghuys et al., 2017). Molecular quantification also enabled the characterization of an intermediate microflora corresponding to a BV-type vaginal microflora with high levels of *G. vaginalis* and *A. vaginae* (Bradshaw et al., 2006; Menard et al., 2008). Cut-off values for *G. vaginalis* and *A. vaginae* might improve BV diagnosis using molecular techniques; however, previous attempts to determine cut-off values most specific and sensitive for the diagnosis of BV have either taken individual species into consideration or a combination of the two species because of the suggested synergism (Redelinghuys et al., 2017). Consensus has not been reached due to variables such as different populations, study design and co-infection status such as HIV and pregnancy. As suggested by Redelinghuys et al. (2017), before a potential cut-off value can be used, it should first be validated with additional sets of data and in different study populations.

Commercially available molecular diagnostic panels for the diagnosis of BV include the detection of the *Lactobacillus* genus, but do not allow an extensive differentiation of species. Current diagnostic panels only allow differentiation of *L. crispatus* and *L. jensenii* (Table 1), but other studies have shown that *L. vaginalis* and *L. iners* also have been found in a population of African women with a low Nugent score (Jespers et al., 2015). In another study *L. vaginalis* was detected in 8% of Belgian women with BV (Jespers et al., 2012). Different species of the *Lactobacillus* genus interact differently with the vaginal environment, which may suggest that each species may contribute to the development of BV through a different mechanism (Jespers et al., 2015).

A VMB dominated by *L. iners* has been characterized as an unstable, transitional VMB and associated with vaginal dysbiosis (Borgdorff et al., 2016; Amabebe and Anumba, 2018; van Houdt et al., 2018). Janulaitiene et al. (2017) found that a healthy VMB was dominated by multiple *Lactobacillus* species, in contrast to BV positive samples where *L. iners* was the most frequently detected species, alone or in combination with *L. crispatus*. Other findings indicated that this particular species of *Lactobacillus* has been found in women with and without BV, indicating the challenge of using *L. iners* as a marker of BV (Zozaya-Hinchliffe et al., 2010).

The same concept may apply to *G. vaginalis* as marker of BV which has four different clades that each possess unique genetic markers and virulence factors (Ahmed et al., 2012). Clades 1 and 3 have been found more commonly among women with BV in contrast to clade 2, which has been associated with an intermediate vaginal microflora (Balashov et al., 2014; Coleman and Gaydos, 2018). Multiple clades have also been associated with BV, suggesting that a polyclonal *G. vaginalis* might be a risk for BV (Balashov et al., 2014). Genotypic differences between *G. vaginalis* strains could explain different clinical phenotypes such as with asymptomatic BV or the detection of *G. vaginalis* in healthy women (Ahmed et al., 2012). It is also of importance to take into consideration that carriers with a dense colonization of *G. vaginalis*, as seen with a high Nugent score, may remain asymptomatic for BV (Nugent et al., 1991; Ahmed et al., 2012). Due to the challenges of using a single species as a marker of BV and the shortcomings of using microscopy for identification of the VMB in BV, characterization of the VMB and its diversity may play an important role in the diagnosis of BV.

A study utilizing 16S rRNA PCR with clone analysis identified greater bacterial diversity in women with BV in contrast to women without BV, which is expected because of complete *Lactobacillus* dominance in a healthy VMB (Fredricks et al., 2005). This molecular technique enabled the identification of 35 unique bacterial species in women with BV by using the bacterium specific 16S rRNA gene (Fredricks et al., 2005). The question remains as to what the clinical significance of the identified species is, because species such as *Atopobium vaginae* and *Gardnerella vaginalis*, were more frequently detected in women with BV while other bacteria such as *Peptostreptococcus* spp., were less frequently detected in BV (Fredricks et al., 2005).

Molecular techniques utilizing the 16S rRNA gene for taxonomic identification also led to the identification of unculturable and fastidious microorganisms such as BV-associated bacterium 1 (BVAB1), BVAB2, and BVAB3 that were found to be specific indicators for BV (Fredricks et al., 2005). This technique also enabled a better understanding of bacterial morphotypes identified using Gram stain analysis (Srinivasan et al., 2013). Molecular amplification and sequencing of the 16S rRNA gene has its limitations, including the introduction of potential bias by broad-range amplification of the bacterial 16S rRNA gene (Winsley et al., 2012; Twin et al., 2013). Differences in taxa between studies can also be attributed to different sequencing approaches and to universal PCR primer mismatches to certain bacteria such as *Bifidobacterium* and *G. vaginalis* (Verhelst et al., 2004). Although the development of molecular techniques has revealed more bacterial species that form part of the pathophysiology of BV, threshold values and the appropriate number of marker bacterial species included on these panels to remain sensitive, specific and cost effective remain to be determined.

### The Potential of High-Throughput Next Generation Sequencing (NGS) for the Diagnosis of BV

Development of a high-throughput technology such as next generation sequencing (NGS) allows the identification of multiple microorganisms that may be present in low abundance in a clinical sample (Hong et al., 2016). Traditional molecular approaches such as targeted Sanger sequencing of the 16S rRNA gene has enabled clinical identification of microorganisms in a polymicrobial sample when compared with traditional culturing methods (Salipante et al., 2013). However, high-throughput sequencing technologies such as targeted metagenomics on an NGS platform have the advantage of simplified identification of multiple microorganisms in a single sequencing approach, compared to interpreting multiple superimposed Sanger sequencing reads (Salipante et al., 2013).

To date, NGS techniques have not been extensively applied in the diagnosis of BV and therefore this section will mainly focus on the potential of the application in the diagnosis and treatment of BV. Next generation sequencing continues to improve the understanding of the complex pathology of BV by enabling the complete and accurate characterization of the diverse VMB associated with BV (Budd et al., 2015). Metagenomic analysis have indicated that bacterial communities associated with BV are highly diverse, either dominated by *G. vaginalis, L. iners* and/or combinations of anaerobic bacteria with or without *Lactobacillus* species in varying proportions (McKinnon et al., 2019). Deep sequencing of the bacterial 16S rRNA gene enabled multiple new bacterial associations with BV, providing new information on the possible role of *Atopobium vaginae* as indicator organism (Fredricks, 2011; Srinivasan et al., 2012; van de Wijgert et al., 2014).

NGS techniques can provide information on the relative proportion and absolute number of all incident bacterial species in the VMB (Ravel et al., 2011; Bostwick et al., 2016). The targeted NGS approach could identify mixed vaginal infections associated with BV and enables a comprehensive investigation of the VMB including the involvement of antibiotic resistance genes (Mullany, 2014). Targeted NGS has been evaluated by Bostwick et al. (2016) as an approach to diagnose and manage BV in clinical practice. The 16S rRNA sequencing approach was evaluated in a case of recurrent BV, where treatment with metronidazole was unsuccessful and a VMB with 56% of anaerobes was identified. In a second case, the patient immediately received treatment with metronidazole based on a positive diagnosis based on Amsel's criteria. The patient developed recurrent BV symptoms with a mixed BV VMB, a *Candida albicans* co-infection and antibiotic resistance. This patient had a VMB dominated by *L. crispatus* and *L. iners*. The use of broad-spectrum antibiotic treatment can induce shifts in the VMB from a healthy VMB often to a *L. iners* transitional VMB (Mayer et al., 2015). In the third clinical case, NGS results indicated a VMB composed of BVAB, *L. iners* and mixed anaerobic bacteria in a patient that was BV positive by Amsel's criteria and with no *G. vaginalis* present. The possibility of mixed bacterial infection is important to consider with the diagnosis of BV. With the NGS approach, the re-emergence of bacterial infection can also be studied, which may aid in the understanding of recurrent BV (Mayer et al., 2015; Bostwick et al., 2016).

Commercial tests that employ cost-effective, short-read 16S rRNA sequencing include the Ion Torrent 16S^TM^ Metagenomics kit (Thermo Fisher scientific, Waltham, MA, USA) in comparison to the Illumina 16S rRNA metagenomic sequencing (Illumina, San Diego, USA), which has been widely used for research purposes (Malla et al., 2019). Next-generation 16S rRNA sequencing has not been utilized in clinical microbiology practice in resource-limited laboratories because of sequencing cost, procedural challenges to prepare the sequencing libraries and the complexity of analysis (Salipante et al., 2013). Various laboratories have evaluated 16S rRNA sequencing for diagnostic purposes and developed analysis pipelines to characterize multiple hypervariable regions of the 16S rRNA gene (Barb et al., 2016; Watts et al., 2017; Culbreath et al., 2019). However, algorithms specifically for the diagnosis of BV is not yet commercially available. Similar to quantitative methods, the cut-off percentage of bacterial abundance related to clinical significance should also be determined. Standardization is needed in terms of the terminology of microbial communities, the methodologies to describe them, and standardization across sample site should also be considered. Targeted sequencing on the NGS platform has similar shortcomings as the 16S rRNA NAATs described earlier. To overcome limited identification of bacteria on species level, long-amplicon PCR-based approaches of the full 16S rRNA gene (~1,500 base pairs) with the Oxford Nanopore Technologies can be considered for accurate characterization of microbial communities and analysis of antibiotic resistance gene islands (Cusc et al., 2018; Malla et al., 2019).

As an alternative to 16S rRNA sequencing, whole genome sequencing (WGS) is another approach to investigate for the diagnosis and management of BV. Whole genome sequencing has the potential to generate a large amount of data from a single isolate, including species, strain type, virulence, antibiotic resistance and other information for outbreak and case management (Besser et al., 2018). Whole genome sequencing has been suggested to be used for microbial epidemiology, the surveillance of pathogens and outbreaks and for the prediction of antibiotic resistance (Jackson et al., 2019). Whole genome sequencing or targeted metagenomics (16S rRNA) might be useful for the diagnosis of BV in clinical cases where an intermediate VMB has been identified [Nugent score finding (score 4–6)]. As an intermediate VMB is proposed to represent a transitional state of the VMB to BV or *vice versa*, NGS techniques can be used to monitor slight changes in terms of abundance and composition of the VMB. The development of NGS technologies is ongoing and is suggested to be highly applicable in the future, where personalized medicine approaches will be possible for the treatment of specific bacterial infections (Punina et al., 2015; Jackson et al., 2019). The personalized medicine approach might also be applicable in BV because of a lack of consensus on the structure and composition of the VMB in this complex condition.

### Metabolomics and Proteomics

Proteomics is the study of the collection of proteins produced by the host and microbiome that embody the functional activity of the entire bionetwork (Peters et al., 2019). Metabolomics is the study of low molecular weight compounds (<1,500 Da) that are produced as substrates or by-products of enzymatic reactions in response to stimuli in a biological system (Peters et al., 2019). Compared to other components in an ecosystem, the metabolome may be the component that best represent the phenotype of that ecosystem (Guijas et al., 2018). Nonetheless, both proteomics and metabolomics are useful in studying host-microbiome interactions on a functional level and could be used to elucidate the pathogenesis of disease (Peters et al., 2019). Newer, high-throughput technologies have created the opportunity to screen and study metabolites and proteins on a large scale. High-throughput mass spectrometry (MS) and nuclear magnetic resonance (NMR) techniques have been used to explore the vaginal milieu under BV conditions and identify proteins or metabolites as biomarkers of this condition (Yeoman et al., 2013; McMillan et al., 2015; Ferreira et al., 2018; Parolin et al., 2018).

Although characteristic for its polymicrobial nature, the BV microbiome is typically associated with the production of specific metabolites, such as cadaverine, putrescine, tyramine and succinate, which have been linked to the “fishy” or amine odor and increased vaginal pH characteristic of BV (Yeoman et al., 2013; McMillan et al., 2015; Pruski et al., 2017). Lactic acid, for example, forms part of the metabolome signature of a healthy VMB (Stafford et al., 2017). Information on the proteome of cervical-vaginal fluid (CVF) is limited, but the protein content of CVF has been shown to differ between a BV microbiome and a healthy VMB (Zegels et al., 2009; Cruciani et al., 2013; Ferreira et al., 2018). Proteins associated with the immune response are either exclusive to BV or show elevated levels of expression (Cruciani et al., 2013; Ferreira et al., 2018). Distinctive metabolites and proteins could therefore be used as biomarkers of a healthy or BV microbiome, irrespective of the dominant bacterial species. A factor which complicates the diagnostic value of metabolomics and proteomics is that it could prove difficult to link the metabolites to the species producing them, which may or may not be essential for treatment purposes.

The evolution of diagnostics research has seen the field shift to evaluating a variety of combination criteria for the detection of BV. Laghi et al. (2014) investigated the microbiome and metabolome of women affected by BV. In a group of women treated with a placebo, this study found that high ratios of lactate to BV-associated metabolites and lactobacilli to BV-associated bacteria were likely to experience spontaneous remission in the absence of treatment, indicating that these may not have been true cases of BV. Another study suggested the coupling of acetate, malonate and nicotinate, quantified with proton (^1^H) NMR, with the VMB species *Atopobium* spp., *M. hominis* and *Prevotella* spp., quantified with qPCR, as diagnostic criteria for BV (Vitali et al., 2015). With the aid of liquid chromatography mass spectrometry (LC-MS), McMillan et al. (2015) have found that higher levels of 2-hydroxyisovalerate (2 HV) and gamma-hydroxy-butyrate (GHB), and reduced levels of lactate and tyrosine, were sensitive and specific for BV. In a validation cohort of 45 pregnant women, 2 HV: tyrosine ratios proved to be the most specific (94%) and sensitive (89%) for BV diagnosis (AUC = 0.946). The combination of a vaginal pH level of more than 4.5 with the detection of specific polyamines has been proposed as diagnostic criteria for BV diagnosis and to assess whether treatment is necessary (Watson and Reid, 2018). The use of high-throughput technologies to screen for biomarkers is still in its infancy (mostly regarding the ease of data analysis) and most of the proposed biomarkers have yet to be validated in different resource-limited settings where they could be implemented as POC tests for BV.

The number of available POC tests that have metabolites or proteins incorporated is limited. The commercial OSOM^®^ BVBlue^®^ test (Gryphus Diagnostics, LLC, Knoxville, TN) is a chromogenic rapid diagnostic test that is based on the detection of activity of the sialidase enzyme, an enzyme that is produced by BV-related bacteria such as *Bacteroides* spp. (Myziuk et al., 2003). Compared to Nugent's criteria, this test has a sensitivity and specificity of around 90% and 96%, respectively; compared to the Amsel criteria the sensitivity and specificity of this test range between 50% and 88%, and between 91% and 100%, respectively (Myziuk et al., 2003; Bradshaw et al., 2005). Although it is reported that a statistically significant correlation exists between a positive BVBlue^®^ test and a raised vaginal pH, it has been found that combining these two criteria for the diagnosis of BV might improve either the sensitivity or specificity of the BVBlue^®^ test, but at the same time could impair the alternative measure (Bradshaw et al., 2005). Another POC test, the FemExam^®^ (Litmus Concepts, Santa Clara, CA) test, is similar to the Amsel's criteria as it measures vaginal pH and amine activity (West et al., 2003). The test is based on a two-card system where card 1 measures vaginal pH and the presence of trimethylamine, where card 2 measures the activity of proline iminopeptidase in *G. vaginalis* (West et al., 2003). West et al. (2003) reported a sensitivity of 91% and a specificity of 62% when the combined test results of the two cards were compared to Nugent's criteria. Both the BVBlue^®^ and FemExam^®^ tests are rapid, easy to perform and objective in comparison to the Amsel and Nugent's criteria. The sensitivity and specificity of these POC tests may be affected or compromised by the polymicrobial nature of BV and the wide spectrum of metabolites and proteins associated with its microbiome. Also, different study populations may harbor varying ratios of species to metabolites and/or proteins, which may or may not be produced in different concentrations.

Direct desorption electrospray ionization mass spectrometry (DESI-MS) analysis could be developed as a POC test as this technique was proven to be successful in identifying mucosal metabolite profiles from swabs that were collected in a clinical setting (Pruski et al., 2017). This technique could be used to explore the mucosal metabolite signature(s) associated with BV and help delineate the bacterial-host interactions at the vaginal mucosa during this condition. Another technology that could be optimized for the POC is low-field benchtop NMR spectroscopy, which involves the use of instruments that operate at frequencies below 100 MHz (Percival et al., 2019). Percival et al. (2019) successfully showed that LF NMR could detect and quantify biomarkers of type 2 diabetes in urine. This study therefore demonstrated the feasibility of the technology to do a biochemical screening of human biofluids.

### Immune System Markers

Although several developing countries rely on the syndromic management of BV based on a vaginal discharge syndrome, Mlisana et al. (2012) reported that vaginal discharge and other symptoms are poor predictors of BV with a sensitivity of 10% and a specificity of 94.4%. This study found that asymptomatic women with one or more active STIs had subclinical inflammation and that these women may have increased levels of genital inflammation similar to those of women with symptomatic infections. Bacterial vaginosis has been associated with a range of upregulated genital pro-inflammatory cytokines (Masson et al., 2014; Kyongo et al., 2015) and women with BV may have an increase in the number of HIV target cells in the genital tract with higher expression levels of immune activation markers (Thurman et al., 2015; Gosmann et al., 2017).

The profiling of immune mediators in the genital tract of different groups of women has revealed variation in these profiles according to age, pregnancy status, gestational age and geographical region (Donders et al., 2003; Kyongo et al., 2015). The polymicrobial nature of BV complicates the assignment of a standard signature of immune mediators to a condition that is associated with divergent microbiome profiles. Different species or microbiome profiles may be associated with different immune mediators and/or different levels of inflammation and the risk of acquiring HIV. For example, *L. crispatus* has been associated with significantly lower levels, and *G. vaginalis, A. vaginae* and *P. bivia* with significantly higher levels, of the cytokines interleukin (IL)-1α, IL-1β and IL-12p70 (Kyongo et al., 2015). Women with a high-diversity VMB profile with abundant *Prevotella* spp. have been reported to have higher levels of interferon (IFN)-γ and IL-1β in cervicovaginal lavages (Anahtar et al., 2015). Also, young women with diverse VMBs dominated by anaerobes other than *Gardnerella* spp. have been reported to have elevated numbers of activated mucosal CD4+ T cells and an over four-fold higher risk of HIV acquisition compared to women with VMBs dominated by *L. crispatus* (Gosmann et al., 2017). In South African adolescent girls aged 16 to 22 years, Lennard et al. (2018) characterized the VMB and found three distinct microbiome subtypes, one of which was associated with extreme genital inflammation. The authors suggested that the inflammatory state associated with the BVAB1-dominated subtype may be chronic and that this subtype can be predicted with a sensitivity of 80% and specificity of 88%, based on a Nugent score of ≥9. The high sensitivity and specificity of high Nugent scores to predict this microbiome subtype might explain why high Nugent scores are representative of certain microbiomes and their associated symptoms but not of all VMB signatures. The Nugent scoring diagnostic system is therefore limited in the sense that it has to represent a complex and diverse VMB in terms of its immune mediator profile and bacterial species dominance. Considering the relationship between the VMB and its immune complement, the immune system is another avenue that could be explored for the development and expansion of BV diagnostics.

Microbiome profiles that represent dysbiosis may not always be detectable with conventional diagnostics. High-throughput technologies have enabled the characterization of the VMB on a grand scale and different bacterial species and microbiome profiles that have been associated with inflammation could therefore be easily characterized and used as indirect markers of an inflammatory state. However, characterizing the microbiome may reveal dysbiosis, but it is not an exhaustive approach to indicate the level of inflammation of the genital tract and therefore the risk of acquiring HIV and other STIs and developing reproductive complications (Mlisana et al., 2012; Masson et al., 2018). Consequently, the polymicrobial nature of BV and its association with divergent immune mediator profiles better support the use of direct markers of inflammation, such as immune mediators, as a diagnostic avenue. Compared with normal microflora, BV is often associated with varying levels of the immune mediators IL-1α, IL-1β, IL-6, IL-12(p70), IL-8, interferon gamma-induced protein (IP)-10, tumor necrosis factor (TNF)-β and secretory leukocyte protease inhibitor (SLPI) (Masson et al., 2014, 2016; Kyongo et al., 2015; Jespers et al., 2017).

In cervical lavage (CVL) samples, Masson et al. (2016) identified that the pro-inflammatory cytokine interleukin (IL)-1β and the chemokine interferon-γ-induced protein (IP)-10 could potentially be used at the POC to identify women with STIs or BV. This study reported that, according to STI and/or BV status, a model that comprised IL-1β (direct relationship between its concentration and BV) and IP-10 (inverse relationship between its concentration and BV) could correctly classify 75% of women with a sensitivity of 77% and a specificity of 72%. With the inclusion of another cytokine, IL-1α (direct relationship between its concentration and BV), the fit of the model was significantly improved (*p* = 0.0001) and the model correctly classified 76% of women with a sensitivity of 72% and specificity of 81%. The performance of the three cytokine biomarkers was validated by Masson et al. (2018) in a cohort of women who were recruited irrespective of vaginal symptoms and who were from four different geographical regions in Africa. The diagnostic model performed best with lateral vaginal swabs as genital specimen, correctly classifying 76% of women based on their STI, BV or intermediate VMB status with a sensitivity and specificity of 86% and 64% respectively. This study found that vaginal pH alone had a low specificity (61%) for classifying women according to their STI or VMB status. However, when vaginal pH was combined with the cytokine biomarkers, the accuracy of the diagnostic test was improved and 82% of cases were correctly identified (sensitivity of 86% and specificity of 64%). Interestingly, the authors also found that by combining pH with only IL-1α, the diagnostic model also correctly classified 82% of cases with a sensitivity and specificity of 86% and 68% respectively. Several other studies support the inverse relationship between IP-10 and BV and the direct relationship between BV and IL-1α and IL-1β (Deese et al., 2015; Kyongo et al., 2015; Jespers et al., 2017).

Immune mediators that are associated with a BV profile could be incorporated into diagnostic tests such as automated chemiluminescent enzyme immunoassays or lateral flow-based immunoassays (POC diagnostic assay). Kunze et al. (2016) measured the concentrations of IL-6 in amniotic fluid and TNF-α in vaginal secretions with the IMMULITE® system (Siemens Healthcare, Erlangen, Germany), an automated chemiluminescent enzyme immunoassay, and the QuickLine rapid test (Milenia Biotec, Gießen, Germany), a lateral flow immunoassay. A comparison between the two assays revealed a strong correlation for each marker with BV, with a Spearman correlation coefficient of 0.88 (*p* < 0.0001) for IL-6 and 0.86 (*p* < 0.0001) for TNF-α. The IMMULITE® system offers sensitive biomarker quantification, including an inflammation panel. The IMMULITE® 1,000 system is a small benchtop immunoanalyzer that is easy to use with a low cost of operation (Zirath et al., 2017). Chaemsaithong et al. (2015) compared the concentrations of IL-6 and IP-10 in amniotic fluid determined by standard enzyme-linked immunosorbent assay (ELISA) and a lateral flow-based immunoassay and reported that the results of the POC test strongly correlated with concentrations as determined by ELISA.

### Computer Algorithm-Based Diagnosis of BV

Although Amsel's criteria and the Nugent scoring system are considered as the “gold standard” for BV diagnosis, problems still exist because of the “interobserver variability” and the fact that the “intermediate vaginal microbiome” do not necessarily indicate disease progression to BV or *vice versa* (van de Wijgert et al., 2014). The advances in machine learning and its application in other fields have been followed by attempts to apply computer algorithms in BV diagnosis (Baker et al., 2014; Beck and Foster, 2014, 2015; Carter et al., 2014; Song et al., 2017; Jarvis et al., 2018).

Computer algorithms could potentially have a wide range of applications that may help clinicians and researchers to search for models that identify features relevant to BV diagnosis, to assess relative bacterial abundance data (qPCR) to diagnose BV, or to analyze bacterial morphotypes on microscope images for more accurate Nugent scoring results (Beck and Foster, 2014, 2015; Carter et al., 2014; Song et al., 2017; Jarvis et al., 2018). One of the first attempts to apply machine learning algorithms in BV diagnosis was done by Beck and Foster (2014), where the authors first grouped the correlations in microbial relative abundance data from studies by Ravel et al. (2011) and Srinivasan et al. (2012) and built different classification models (based on Amsel's criteria or Nugent scoring) using three different types of machine learning algorithms [“genetic programming” (GP), “logistic regression” (LR) and “random forest” (RF)]. The classification models using RF and LR obtained accuracy values between 90% and 95% when the models classified potential diagnostic features and bacterial groups associated with Nugent scoring, whereas the models using GP obtained slightly lower accuracy values (above 80%; Beck and Foster, 2014). However, the identified diagnostic features differed between all three machine learning algorithms and a technical difficulty was encountered when the models were deconstructed to identify which diagnostic features influenced the classification accuracy (Beck and Foster, 2014). These observations led to a follow-up study in 2015 where the changes in the accuracy values change were observed when each diagnostic feature was sequentially added to the classification models created by RF and LR (Beck and Foster, 2015). Instead of grouping the VMB according to microbial relative abundances, features were all ranked according to their importance in each classification model (Beck and Foster, 2015). The results revealed that a relatively consistent decrease in classification accuracy was observed as the RF classification model feature ranking decreases, while a more uneven decrease was observed for the LR model feature rankings, suggesting that the RF classification may be more useful in predicting BV (Beck and Foster, 2015). The top 15 important features for BV classification models based on Nugent scoring included several BV-associated bacteria (*Prevotella* spp., *G. vaginalis, Dialister* spp., *A. vaginae, Megasphaera* spp., *Eggerthella* spp., *Sneathia* spp., *Peptoniphilus* spp.) and clinical features like vaginal pH and “clue cells” (Beck and Foster, 2015).

Computer algorithms can also be incorporated into a molecular diagnostic tool to assess the relative abundance data of BV-associated bacteria for diagnosing BV. In a recent study conducted by Jarvis et al. (2018), a novel qPCR-based diagnostic tool (that detects ten BV-associated bacterial species and four *Lactobacillus* species) with a diagnostic machine learning algorithm (CLS2.0q) was developed. Evaluation of this molecular diagnostic tool with 172 women (149 symptomatic and 23 asymptomatic) resulted in a sensitivity of 93% and a specificity of 90% when compared with Nugent scoring (Jarvis et al., 2018). Although this molecular diagnostic tool currently requires further validation and improvements to become useful in clinical settings, it could be handy in POC settings.

Lastly, computer-based algorithms could be used to automate BV diagnosis based on microscopy results. In a study by Song et al. (2017), different approaches (“Bacteria Regions Segmentation,” “Overlapping Bacteria Clumps Splitting” and “Bacteria Morphotypes Learning”) were introduced to automate BV diagnosis based on the Nugent scoring system. The automated BV diagnosis procedures were as follows: (i) the selection of images from a slide; (ii) the generation of the superpixels by grouping pixels of different regions of bacteria; (iii) the calculation of superpixel contrast at the region level; (iv) the segmentation of “bacteria regions” by “saliency cut”; (v) the splitting of overlapping bacterial clumps; (vi) the classification of bacterial morphotypes through a machine learning algorithm; and (vii) BV diagnosis using the Nugent scoring system (Song et al., 2017). The experimental results using 105 vaginal smears revealed a sensitivity of 58.3% and a specificity of 87.1%, and the authors presumed the reason for low sensitivity was due to an inability of the algorithm to split heavily overlapped bacteria (Song et al., 2017). However, when the images were manually selected by human readers (experts) before the superpixel generation, sensitivity improved by up to 75% (Song et al., 2017). Again, this approach requires more research and improvements before it can be used in clinical settings, but it could be beneficial in future as it provides a more objective analysis of the vaginal smears and prevents “interobserver variability.”

## The Role of the Polymicrobial Biofilm in the Treatment and Diagnosis of BV

Bacterial vaginosis can be diagnosed microscopically with the presence of “clue cells,” one of the four components of Amsel's criteria, but it was only in 2005, when Swidsinski et al. (2005) described the adherent biofilms found on these squamous vaginal epithelial cells. A spatial organization of bacteria associated with the vaginal epithelium shed new light on the etiology of BV but also led to the definition of BV as a synergistic polymicrobial syndrome with not only *G. vaginalis* playing an important role (Holst, 1990; Sobel, 2000; Swidsinski et al., 2005). Bacterial vaginosis is associated with a polymicrobial biofilm formed by BV-associated *G. vaginalis* and other BV-associated bacteria including *A. vaginae, Mobiluncus mulieris* and *Prevotella bivia* (Castro and Cerca, 2015; Castro et al., 2016, 2019; Hardy et al., 2016). This polymicrobial biofilm might be one of the reasons for the high recurrence rate of BV due to the protection of bacteria against H_2_O_2_, lactic acid, bacteriocins and antibiotics commonly used for treatment (such as metronidazole and tinidazole), but also provides resistance against the host immune system (e.g., prevent macrophage phagocytosis or chemotaxis; Al-Mushrif et al., 2000; Patterson et al., 2007; Alves et al., 2014; Swidsinski et al., 2015; Castro et al., 2019).

Biofilms composed mainly of *G. vaginalis* have the ability to adhere to vaginal epithelial cells, even in the presence of *L. crispatus*, and have also shown to benefit from the colonization of other BV-associated bacteria (*A. vaginae, M. mulieris, P. bivia, Fusobacterium nucleatum*; Machado et al., 2013a,b; Gottschick et al., 2017). Moreover, the adherence of *G. vaginalis* has been found to increase in the presence of *L. iners*, which could be due to the weak protective action exhibited by *L. iners*, although *L. iners* and *L. crispatus* have shown similar inhibitory effects against the adherence of BV-associated bacteria (Machado et al., 2013b). This investigation on the adherence of *L. iners* and *G. vaginalis* on vaginal epithelial cells confirmed that *L. iners* does not exhibit an antagonistic effect against *G. vaginalis* and suggested that the two species may be tolerant toward each other in the vaginal environment (Machado et al., 2013b). Such findings are important to fully understand biofilm formation and structure if the investigation thereof would be used for diagnostic and monitoring purposes and might especially be important in the adjustment of treatment in cases of a high recurrence rate.

*Gardnerella vaginalis* is known as the initial colonizer in the biofilm formation in BV, accounting for 60% of the bacterial composition. Initial colonization involves the attachment and formation of a biofilm (Machado and Cerca, 2015; Hardy et al., 2016). Transcriptomic analysis of *G. vaginalis* gene expression (e.g., virulence genes such as vaginolysin and sialidase) in biofilms indicated gene-regulated processes to result in a protected form of bacterial growth with high virulence and low metabolic activity (Castro et al., 2017, 2019). Gene expression of *G. vaginalis* transcripts encoding antimicrobial resistance proteins might be of particular interest, since a polymicrobial biofilm has been suggested to have greater antibiotic tolerance in contrast to a mono-species biofilm (Castro et al., 2019). This phenotype of *G. vaginalis* is another mechanism that may contribute to the recurrent nature of BV and may be a potential biomarker for biofilm formation and potential target for treatment remedies; however, extensive investigation on how such gene expression is influenced still needs to be done.

Both *G. vaginalis* and *A. vaginae* are accepted as important bacterial constituents of the BV biofilm, although single colonization of *A. vaginae* has not been found to initiate biofilm formation. Research findings highlighted higher bacterial loads when both species were present in a biofilm, in contrast to biofilms of only *G. vaginalis*, highlighting the synergistic relationship of these two species in biofilm formationto create favorable conditions for optimal growth and survival (Hardy et al., 2015). Such a synergistic relationship could also play a role in increased antibiotic resistance in BV and it has been demonstrated that different *A. vaginae* strains have different susceptibility profiles *in vitro* to metronidazole and secnidazole (Mendling et al., 2019). With the known increased recurrence rate of BV, antiseptics such as dequalinium chloride (DQC) (Fluomizin®) has been accepted as an alternative treatment for BV. This compound has antimicrobial activity against different pathogens (aerobic and anaerobic bacteria), especially against *G. vaginalis* and *A. vaginae* and has no known safety concerns and mechanisms of resistance (Weissenbacher et al., 2012).

Microbial communities, such as the VMB, is known as reservoirs for numerous antimicrobial resistance genes. Collectively these genes harbored in a microbial community is called the resistome, which can be studied by means of functional metagenomics and targeted (PCR-based) metagenomics, including real-time PCR and sequence-based metagenomics (Penders et al., 2013). Characterization of the resistome allows the investigation of present antimicrobial resistance (AMR) genes in a microbial environment, which might be useful in the investigation of the VMB and biofilms in BV for optimal treatment remedies. Although resistome research has mostly been done with the gut microbiome, and only aerobic biofilm samples of wastewater have been tested, the resistome might be an area of research that may give more insight into antibiotic resistance associated with BV (van Schaik, 2015; Relman and Lipsitch, 2018; Tian et al., 2019).

Another area that may be relevant to investigate in BV is microbiome-drug interactions, more recently termed pharmaco-microbiomics. In pharmaco-microbiomics, individual variation in drug response can be addressed by characterizing the composition of microbial communities and identify the chemical mechanisms in these microbiomes to understand drug metabolism (Guthrie and Kelly, 2019). A relationship has already been established between the VMB and levels of antiretrovirals (ARVs), for instance, in women with a VMB consisting of *G. vaginalis* and other anaerobic bacteria lower levels of tenofovir were detected than in women with a *Lactobacillus-*dominating microbiome (Donahue Carlson et al., 2017; Klatt et al., 2017). The finding was that a non-*Lactobacillus* microbiome rapidly depleted tenofovir before target cells could actively convert it to a pharmacologically active drug (Klatt et al., 2017). Therefore, tenofovir would be less effective in women with a non-*Lactobacillus* or BV type of microbiome. Besides the investigation of the metabolism of ARVs by microbial communities, the question is if such an effect would be suspected with the treatment of BV. Research regarding the metabolism of metronidazole or other antimicrobial drugs by the VMB has not been done to establish the efficacy of drugs with a BV type of microbiome. Moreover, research regarding the metabolism of drugs by biofilms in BV might contribute to additional treatment remedies or alteration of current treatment remedies such as initial drug concentration and mode of delivery.

For an optimal treatment approach for BV, the composition and structure of a biofilm can be investigated by using the fluorescence *in situ* hybridization (FISH) method followed by visualization with fluorescence microscopy, as described by Swidsinski et al. (2005) and Machado et al. (2013a). The presence and adherence of both *G. vaginalis* and *A. vaginae* can be investigated by using peptide nucleic acid fluorescence *in situ* hybridization (PNA FISH), which includes a specific PNA probe specifically selecting for the bacterial species of interest followed by DAPI staining to quantify cells in a mono- and dual-species biofilm (Freitas et al., 2017; Castro et al., 2019). A combination of confocal laser scanning microscopy (CLSM) and FISH has been recommended by Castro et al. (2019) to investigate the spatial distribution of a bacterial population and different architectures of the tested dual-species biofilms. Hardy et al. (2015) developed a PNA probe specific for *A. vaginae* (AtolTM1 probe), with a 67% sensitivity and 89.4% specificity as tested on clinical samples as compared to quantitative PCR (qPCR). The PNA FISH and qPCR can be used to determine the bacterial load of *G. vaginalis* and *A. vaginae* on vaginal epithelial cells, which is relevant in the prediction of the presence of a bacterial biofilm in BV (Hardy et al., 2015). Methods such as PNA FISH may be useful in cases of recurrent treatment failure, where it can be used to detect changes in biofilm composition and simultaneously monitor treatment efficacy. This method may therefore rather form part of a personalized treatment or precision medicine approach, especially in cases of high recurrence of BV with unsuccessful attempts of depletion of the biofilm structure in BV, and not as a method for standard diagnostic purposes.

## Conclusion

The polymicrobial nature of BV necessitates the use of diagnostic tests that are based on combination criteria. Part of the challenge lies in determining which combination criteria are sensitive and specific enough as diagnostic criteria for BV; the other challenge is to develop cost-effective diagnostic tests, which could preferably be used at the POC. Although there is a complex interplay between vaginal pH and the concentration of different bacterial species, it is evident that vaginal pH, specifically higher than 4.5, improves the performance of diagnostic tests when combined with other components in CVF.

The efficient and accurate detection of vaginal dysbiosis has always been plagued by factors such as the difference in biomarker levels across populations (e.g., bacterial species) and sample type variations (Kyongo et al., 2015; Masson et al., 2018). Many studies discussed in this review have highlighted the potential of different combination criteria with biomarkers beyond the genetic level to improve BV diagnostics. If the purpose of diagnosis is to treat, the question should be asked whether a perfect equilibrium exists between the VMB and all its related components. That is, does bacterial concentration translate into corresponding levels of metabolites, proteins and inflammatory markers? Nonetheless, the continual reduction in operational costs of high-throughput technologies provide the opportunity to study the vaginal milieu with a systems biology approach on a large scale to map and link potential biomarkers. This review does not necessarily suggest the replacement of diagnostic tools currently available for BV but does highlight the limitations of these tools and calls for the expansion of the BV diagnostics field by exploring the vast array of diagnostic opportunities discussed here.

In many resource-limited settings, however, POC tests for BV are either not available or simply too expensive for routine diagnostic use and healthcare practitioners have to rely on syndromic management of vaginal discharge syndrome. It is therefore imperative that the development and evaluation of new diagnostic tests must include both a cost- and health-benefit analysis in various settings, especially where expensive instrumentation is required. The risk profiles of different populations for adverse sequelae of BV infection, such as increased risk for HIV infection and poor pregnancy outcomes should guide diagnostic test selection. In such at-risk populations, we have to ask the question—what is the cost of cost?

## Author Contributions

All authors contributed to concept design and layout of the manuscript. MR, JG, and HJ wrote the body of the manuscript and MK contributed as senior author. All authors listed approved the manuscript for publication.

## Conflict of Interest

The authors declare that the research was conducted in the absence of any commercial or financial relationships that could be construed as a potential conflict of interest.
